# Correction: Phylogenetic Properties of 50 Nuclear Loci in *Medicago* (Leguminosae) Generated Using Multiplexed Sequence Capture and Next-Generation Sequencing

**DOI:** 10.1371/journal.pone.0127130

**Published:** 2015-05-01

**Authors:** The PLOS ONE Staff

## Notice of Republication

The article was republished on April 14, 2015 to include a reference that was missing from the published article. The reference should be: [11] Thomson RC, Wang IJ, Johnson JR (2010) Genome-enabled development of DNA markers for ecology, evolution and conservation. Molecular Ecology 19: 2184–2195.

## Supporting Information

S1 File Originally published, uncorrected article. (PDF)

S2 File Republished corrected article. (PDF)
