# Supplementary material for: Phylogenetic Properties of 50 Nuclear Loci in Medicago (Leguminosae) Generated Using Multiplexed Sequence Capture and Next-Generation Sequencing
Source: PLoS One. 2014 Oct 17;9(10):e109704. doi: 10.1371/journal.pone.0109704 (PMC4201463; doi:10.1371/journal.pone.0109704)
Supplement: S1 File — Figure B, Calibrated BEAST trees of gene 9 showing posterior probability values for each node. Figure C, Calibrated BEAST trees of gene 48 showing posterior probability values for each node. Figure D, Calibrated BEAST trees of gene 50 showing posterior probability values for each node. Methods A, Additional methods. Table A, Reads per sample before and after adapter removal and quality trim (phred score threshold of 20). (PDF) [file pone.0109704.s002.pdf]

## Supplementary Material

**Table A: reads per sample before and after adapter removal and quality trim (phred score threshold of 20)**

| sample | total reads | reads kept after quality trimming | % reads kept |
|---|---|---|---|
| *Medicago italica* | 603490 | 529164 | 88% |
| *Medicago sativa* | 852870 | 620087 | 73% |
| *Medicago medicaginoides* | 689712 | 606195 | 88% |
| *Melilotus neapolitanus* | 1107464 | 909632 | 82% |
| *Melilotus sulcatus* | 1622968 | 1192313 | 73% |

### Methods A

Sequences of four additional species of *Medicago* (*M. littoralis*, *M. papillosa*, *M. marina* and *M. prostrata*) were produced following the protocol presented in the Methods section. Multiple individuals were sampled for *M. papillosa* and *M. prostrata,* in the latter case using mostly herbarium preserved specimens. Following sequence assembly, the program CLC-mapper (CLC Bio, Aarhus, Denmark) was used to map reads to the assembled sequence of genes 7 (Medtr2g038270.1), 9 (Medtr3g079830.1), 48 (Medtr3g113790.1) and 50 (Medtr3g113960.1). From the mapped reads, alleles were phased using the program Samtools phase (Li et al., 2009) and phased reads were re-assembled into contigs using CLC-assembler and aligned in Geneious Pro (v.5.3.6). Model testing was done using the reversible-jump

mcmc implemented in MrBayes v.3.1.2 (Huelsenbeck and Ronquist, 2005) and gene trees were reconstructed in BEAST v1.8.0. (Drummond and Rambaut, 2007).

**Supplementary figure legends:**

Calibrated BEAST trees of genes 7 (Figure A), 9 (Figure B), 48 (Figure C) and 50 (Figure D) showing posterior probability values for each node.

**Supplementary figures:**

**Figure A**

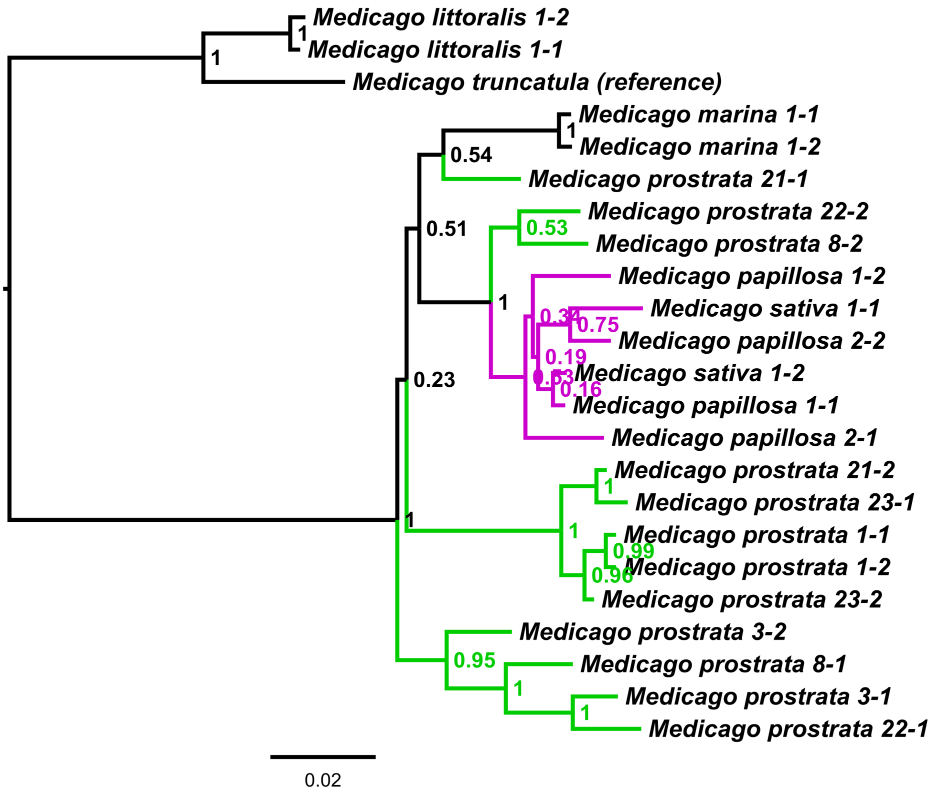

## Figure B

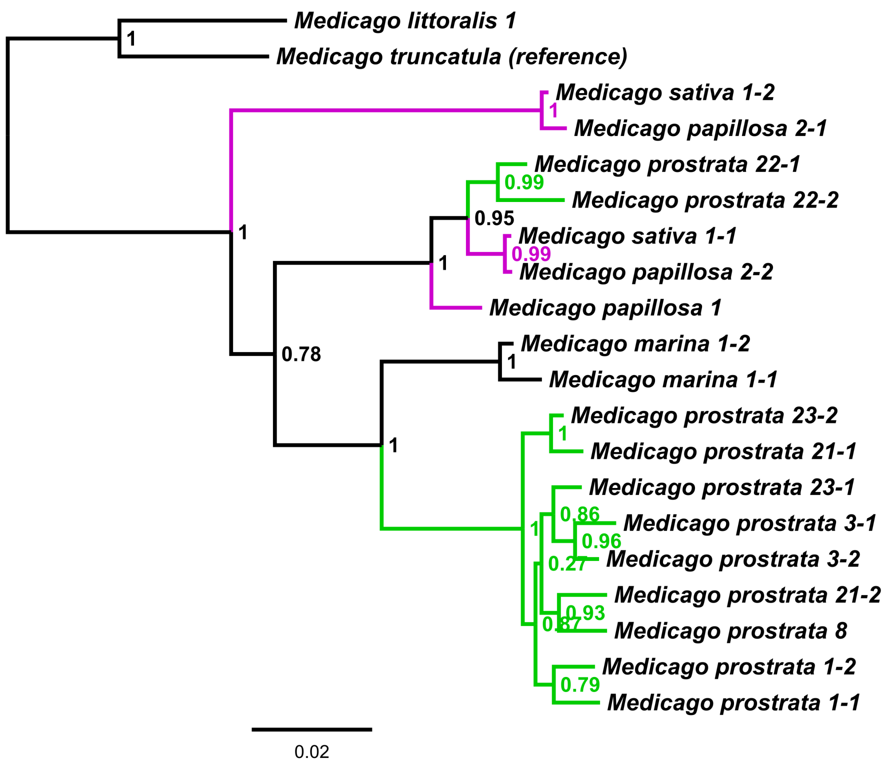


## Figure C

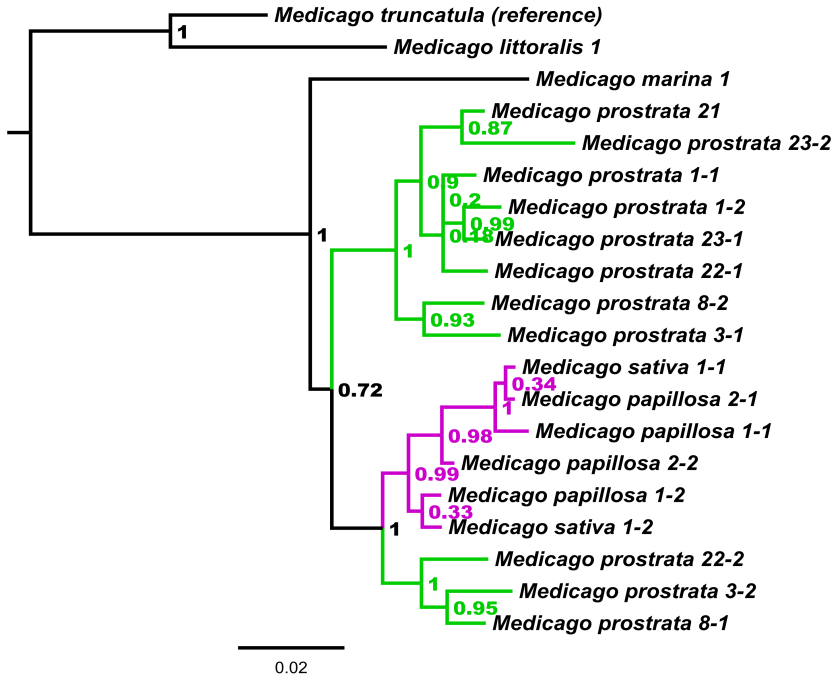

**Figure D**

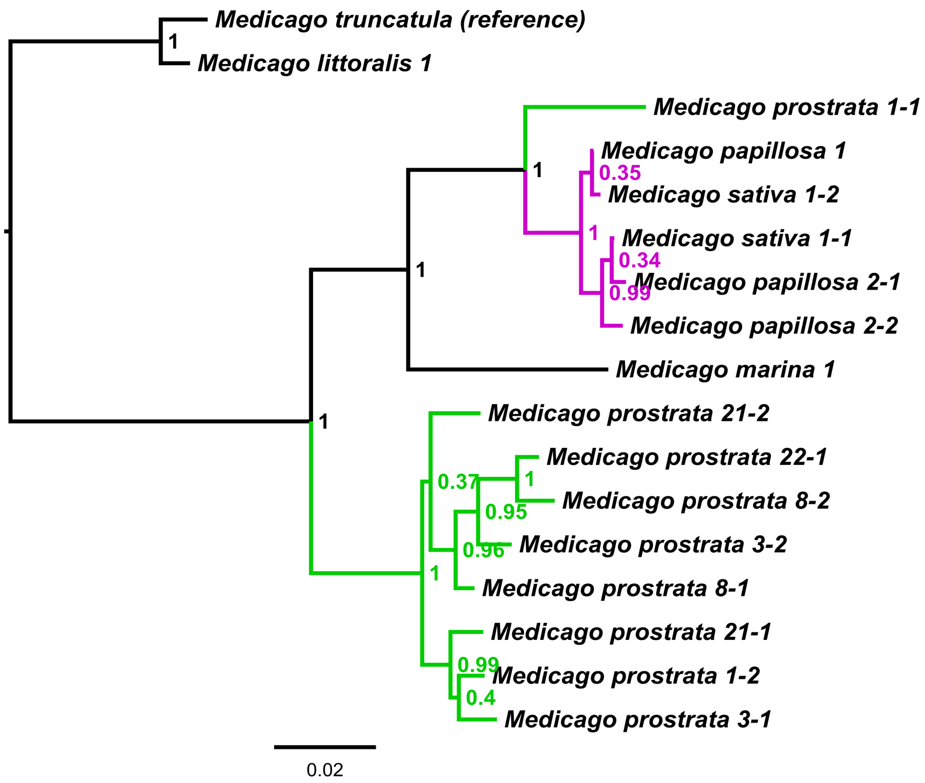
Medicago truncatula (reference)
1
Medicago littoralis 1
Medicago prostrata 1-1
Medicago papillosa 1
0.35
Medicago sativa 1-2
1
Medicago sativa 1-1
1
0.34
Medicago papillosa 2-1
0.99
Medicago papillosa 2-2
1
Medicago marina 1
Medicago prostrata 21-2
1
Medicago prostrata 22-1
0.37
1
Medicago prostrata 8-2
0.95
Medicago prostrata 3-2
0.96
1
Medicago prostrata 8-1
Medicago prostrata 21-1
0.99
Medicago prostrata 1-2
0.4
Medicago prostrata 3-1
0.02
