# Supplementary material for: Correction: Phylogenetic Properties of 50 Nuclear Loci in Medicago (Leguminosae) Generated Using Multiplexed Sequence Capture and Next-Generation Sequencing
Source: PLoS One. 2015 May 1;10(5):e0127130. doi: 10.1371/journal.pone.0127130 (PMC4416814; doi:10.1371/journal.pone.0127130)
Supplement: S2 File — (PDF) [file pone.0127130.s002.pdf]

PLOS | ONE

RESEARCH ARTICLE

Phylogenetic Properties of 50 Nuclear Loci in *Medicago* (Leguminosae) Generated Using Multiplexed Sequence Capture and Next-Generation Sequencing

Filipe de Sousa[1]*, Yann J. K. Bertrand[1], Stephan Nylinder[2], Bengt Oxelman[1], Jonna S. Eriksson[1], Bernard E. Pfeil[1]

1 Department of Biological and Environmental Sciences, University of Gothenburg, Gothenburg, Sweden,
2 Department of Botany, Swedish Museum of Natural History, Stockholm, Sweden

* filipedeportugal@gmail.com

OPEN ACCESS

Citation: de Sousa F, Bertrand YJK, Nylinder S, Oxelman B, Eriksson JS, Pfeil BE (2014) Phylogenetic Properties of 50 Nuclear Loci in *Medicago* (Leguminosae) Generated Using Multiplexed Sequence Capture and Next-Generation Sequencing. PLoS ONE 9(10): e109704. doi:10.1371/journal.pone.0109704

Academic Editor: Sven Buerki, Royal Botanic Gardens, Kew, UNITED KINGDOM

Received: May 6, 2014

Accepted: September 10, 2014

Published: October 17, 2014

Copyright: © 2014 de Sousa et al. This is an open access article distributed under the terms of the Creative Commons Attribution License, which permits unrestricted use, distribution, and reproduction in any medium, provided the original author and source are credited.

Data Availability Statement: All relevant data are within the paper and its Supporting Information files and all sequence files are available at the European Nucleotide Archive with accession numbers ERS511665, ERS511666, ERS511667, ERS511668, ERS511669.

Funding: This work was supported by grants from the Swedish Research Council, the Royal Swedish Academy of Sciences (grant 2009-5206), Lars Hiertas Minne fund, The Royal Physiographic Society in Lund, Helge Ax:son Johnsons fund, and the

Abstract

Next-generation sequencing technology has increased the capacity to generate molecular data for plant biological research, including phylogenetics, and can potentially contribute to resolving complex phylogenetic problems. The evolutionary history of *Medicago* L. (Leguminosae: Trifoliae) remains unresolved due to incongruence between published phylogenies. Identification of the processes causing this genealogical incongruence is essential for the inference of a correct species phylogeny of the genus and requires that more molecular data, preferably from low-copy nuclear genes, are obtained across different species. Here we report the development of 50 novel LCN markers in *Medicago* and assess the phylogenetic properties of each marker. We used the genomic resources available for *Medicago truncatula* Gaertn., hybridisation-based gene enrichment (sequence capture) techniques and Next-Generation Sequencing to generate sequences. This alternative proves to be a cost-effective approach to amplicon sequencing in phylogenetic studies at the genus or tribe level and allows for an increase in number and size of targeted loci. Substitution rate estimates for each of the 50 loci are provided, and an overview of the variation in substitution rates among a large number of low-copy nuclear genes in plants is presented for the first time. Aligned sequences of major species lineages of *Medicago* and its sister genus are made available and can be used in further probe development for sequence-capture of the same markers.

Introduction

The development and rapidly growing capacity of next-generation sequencing (NGS) has greatly increased the amount of data generated for research in plant biology. Large datasets of molecular sequences are now being collected across various model and non-model organisms by sequencing whole genomes, transcriptomes, or through enrichment of multiple genes at

Lundgrenska fund to B.E.P.; from the P. A. Larssons fund and Lars Hiertas Minne fund to F.S. The funders had no role in study design, data collection and analysis, decision to publish, or preparation of the manuscript.

Competing Interests: The authors have declared that no competing interests exist.

either specific or anonymous loci [1]. Systematic biology is also set to benefit from these developments, with several projects having already used NGS to obtain data [2–5]. However, the application of NGS in phylogenetics is still in its infancy and far from routine, partly because there has been no consensus on the choice of sampling strategy [6].

Whole genome sequencing has been used to explore individual variation at the genomic level in plants [7–8] but, due to its high price, is not expected to be widely applied for plant phylogenetic research in the near future. Anonymous locus approaches, such as restriction-site-associated (RAD) tags [9] have been successfully used to solve species relationships [3], [10], but do not always result in good overlap among samples, which may compromise the overall cost efficiency of these methods. Furthermore, anonymous loci are likely to have higher levels of paralogy and a short phylogenetic span [11]. Genome skimming approaches [12] can be used to sequence the high-copy fraction of plant genomes (cpDNA, mtDNA, rDNA) and, to some extent, to identify nuclear loci, but in the latter case the amount of information obtained is limited and highly dependent on sequencing depth and genome size.

Hybridisation-based enrichment (or sequence capture), on the other hand, appears to have great potential to solve these challenges by selecting, *a priori*, loci of interest, or those that have suitable parameters for analysis, to generate larger and more informative data sets if compared to other genomic sampling strategies [13]. Sequence-capture has already been used in phylogenetics and phylogeography, in both plants and animals [2], [4–5], [14–17] and is likely to replace PCR as the main target enrichment method in plant sciences [1], [18]. One or more genomes or transcriptomes are necessary for probe design prior to sequence-capture, but for groups where a close reference is lacking, protocol modifications can be made to capture targets that are not phylogenetically close to the reference [19]. Hybridisation-based enrichment can also overcome the problem of degraded genomic DNA, which is often encountered in herbarium and museum material [20–21]. Multiplexing of indexed DNA libraries for sequence-capture significantly reduces the amount of work and time required to obtain the same data via PCR amplification of the target, while also reducing sequencing costs when combined with NGS platforms such as Illumina [22]. Multiplexing requires that the size of the target is not excessive, otherwise the read depth (number of reads at a particular site) might be insufficient for proper contig assembly and variant calling. Furthermore, keeping the targets to moderate sizes while generating longer sequences (long loci rather than SNP/Rad-tag data) produces more informative data per locus. Generating large alignments may imply a significant amount of manual work, but enables the inference of more resolved and robust gene trees and consequently the correct assessment of gene tree incongruence, for which SNP and Rad-tag data are severely limited. The cost per base of sequence is vastly lower in NGS than in Sanger sequencing [23] but the overall investment, especially for sample preparation, is still considerable. Therefore, instead of relying solely on exploratory sampling of new loci, it is worth also considering sampling characterised markers that have already been tested for both ease of recovery with sequence capture methods and suitable sequence variability. Targeting previously employed loci is especially important in phylogenetics and phylogeography, which require homologous molecular data from multiple individuals [6], because newly produced sequences can easily be incorporated into pre-existing phylogenies. As more researchers use the same loci across many taxa, large phylogenies can be inferred using data sets with much lower proportions of missing data than is typically the case at present (e.g., 93–95% in [24–25]).

Medicago L. (Leguminosae: Trifoliae) comprises c. 87 species of annual and perennial herbs and shrubs [26] that diverged within about the last 10 Ma ([27], Pfeil *et al.* unpublished data). Several species are important global forage and green manure crops, but are also grown for soil nitrogen-fixation and human consumption [26]. Attempts have been made to infer a species phylogeny of the genus based on molecular data, using chloroplast [28–29], mitochondrial [30]

and nuclear genes [31–33], [29–30], as well as SNP data collected across the genome [34], but despite this apparent abundance of data, the evolutionary history of *Medicago* is still poorly understood due to significant incongruence among (or within, in the case of the SNPs) the phylogenies produced thus far. To understand the causes of genealogical conflict among different portions of the *Medicago* genome and infer a correct species phylogeny, more molecular data must be obtained across the genus, preferably for low-copy nuclear (LCN) genes, which are expected to be sufficiently informative to produce well-resolved gene trees [35]. In comparison with organellar cpDNA, some LCN gene sequences can have higher rates of evolution and can produce better supported gene trees [36–37], although these rates may vary dramatically [37–38]. The fact that LCN genes have bi-parental inheritance facilitates the identification of reticulation and likely parental lineages in cases of hybridisation and polyploidy [39]. LCN genes also allow for the combination of multiple independent (unlinked) loci, which provide a broader representation of the underlying processes shaping phylogenetic tree topologies than single or linked loci, such as those from cpDNA; if compared to high copy or highly repetitive regions of the chromosomes, LCN genes tend to be more stable in both position and copy number [39]. Nuclear ribosomal dna (rDNA) is often employed in phylogenetics, but regions such as ITS show significantly higher homoplasy than other DNA regions [40], which may mislead phylogenetic inference if the homoplasy is due to incomplete concerted evolution (i.e., potentially leading to a mix of characters with different histories). LCN genes are often employed in phylogenetics but little information about their properties is available (e.g., [37]). Prior knowledge of substitution rates, for instance, can significantly contribute to improve the inference of phylogenetic trees, especially in a Bayesian framework. Studies in *Gossypium* [38–39] showed a significant range of sequence variation among nuclear genes, but so far no studies have explored the variation of nucleotide substitution rates across a large sample of nuclear genes.

It should be noted that the two main functional partitions of LCN genes, namely exons and introns, provide a strong starting point for many analytical decisions when inferring phylogenies. For deeper (older) questions, exon sequences will be more easily aligned, not likely to be saturated and perhaps better described by current models of substitutions than intron sequences. Even when third positions are saturated, the remaining positions can carry conservative information to anchor the more homoplasious third positions, thereby together generating robust estimates [41]. However, for shallow (young) questions (among closely related species), exons can be conservative to the point of invariance, making intron sequences extremely important in this context. Even where exons carry some information for shallow questions and might be used alone for phylogenetic inference, as the length of the sequence required increases, so does the likelihood of intra-locus recombination (because genetic and physical distance are correlated). For primer and probe design, exon sequences are highly useful to capture intron sequence information, so LCN genes containing introns are a rich resource to be exploited. It should be noted that, although modelling and alignment may appear to give exons the edge for moderately deep questions, lineage-specific shifts in function and rate for specific sites can cause misleading results [42–43], even when analysing closely related genera, but may not necessarily affect adjacent non-coding sequences(e.g., [44]).

Here we report on 50 new markers developed for phylogenetics in *Medicago* L. using the genomic resources of the *M. truncatula* Gaertn. genome project [45] and hybridisation-based gene enrichment. We use six representative species of *Medicago* and its sister genus *Melilotus* (= *Trigonella*, [28]) to assess the variation among sequences and the phylogenetic utility of each locus. We estimate substitution rates for all 50 genes from the corresponding gene trees and explore the variation in substitution rates among these LCN. Our probe set and alignments are made available as a resource for sequence-capture in *Medicago* and related genera.

Materials and Methods

Choice of candidate genes

The genome of *Medicago truncatula* [45] was scanned for genes with potential phylogenetic utility. Our sample consisted of 62 LCN genes divided into 20 groups of three to four tightly linked genes. The sampling scheme derives from the assumption that unlinked loci provide information on different evolutionary histories within a group of organisms (e.g., due to hybridisation), whereas tightly linked genes tend to follow the same evolutionary history. Genes were chosen according to the following criteria and parameters: distance between each linked gene within groups < 30Kbp, length of genes ≥ 2Kbp; introns ≤ 500bp, genes single-copy within the *M. truncatula* genome, genes with homologues in other genomes (e.g., *Lotus* L., *Glycine* Willd., *Populus* L., *Arabidopsis* Heynh.), genes expressed in any part of *M. truncatula*. The number of copies of the selected sequences was assessed using BLAST with a threshold E-value of 1E-12. Genes were either sampled entirely or in part, depending on the respective size. In addition to our 62 genes, we sampled 257 short loci (150–200 bp exon sequences used for probing) distributed across all *Medicago* chromosomes, thus increasing the target size to 185 Kb, but these short loci will not be dealt with herein.

Sampling and library construction

DNA from accessions of *Medicago*, *Melilotus* and *Trigonella* (tribe Trifoliae, subtribe Trigonellinae) was extracted from silica dried leaf tissue and herbarium specimens using the Dneasy Plant Mini Kit (Qiagen, Valencia, CA, USA). Genomic DNA was sheared with a Covaris S220 instrument (Covaris, Woburn, Massachusetts, USA). A DNA library was constructed with the NEXTflex DNA Sequencing Kit and NEXTflex Barcodes (BIOO Scientific, Austin, Texas, U.S.A.). Agencourt AMPure XP magnetic beads (Beckman Coulter) were used for fragment size selection to exclude fragments under 300 bp and over 400 bp, and also for DNA purification. For better yield, a PCR run of 14 cycles was performed on the indexed samples, using the reagents provided with the kits, with the following program: 98°C, 2'; 14x(98°C, 30"; 65°C, 30"; 72°C, 60"); 72°C, 4'. Concentrations of the amplified library were measured in a NanoDrop 2000c instrument (Thermo Fisher Scientific,Waltham, MA, USA).

Gene enrichment and sequencing

Gene enrichment was carried through the MYBaits target enrichment system (MYcroarray, Ann Arbor, Michigan). Probes were designed with a 3x tiling density (90-mer probes tiled every 30 bp) from the FASTA sequences of each gene. The reference genome annotation displayed the expected position of exons and introns, but for probe design, the whole sequence of the gene was used. Given the modest size of the target relative to the capacity of the enrichment kit, and the fact that the genes chosen are present in single copy number in the reference genome, the inclusion of intron sequences in probe design was considered justified. Lemmon *et al.* [22] showed that long fragments captured by exon-based probes could also cover introns. In our sampling, genes with small introns (< 500 bp) were preferred, so that if probes based on intron sequence failed to capture fragments, probes based on the adjacent exons would still capture fragments covering the entire intron.

Equimolar amounts of each amplified library were pooled in reactions of eight indexed samples each. Preliminary tests comparing pooling of six and 12 *Medicago* samples in the same sequence capture reaction suggested that pooling of eight individuals was the best compromise in terms of efficiency and read depth. Individuals were also pooled according to phylogenetic proximity suggested by previously published phylogenetic trees; for example, all *Melilotus* and

Trigonella samples were pooled in the same reaction, as the former is nested in the latter [28–29]. Prior to gene enrichment, DNA was concentrated using a SpeedVac (Savant Instruments, Farmingdale, USA).

Hybridisation reactions were performed at 65°C for 36 hours as indicated in the standard MYBaits protocol. Probes were recovered with Dynabeads MyOne Streptavidin C1 (Invitrogen Dynal AS, Oslo, Norway). To obtain a higher concentration of DNA for sequencing, a PCR run of 14 cycles was performed for each hybridisation reaction using Herculase II Fusion DNA Polymerase (Agilent, Waldbronn, Germany) and the following program: 98°C, 30''; 14x(98°C, 20''; 60°C, 30''; 72°C, 60''); 72°C, 5'. High-throughput 150 bp paired-end reads were produced for a total of 48 mutiplexed samples on a MiSeq platform from Illumina (San Diego, California, USA) at the Genomics Core Facility of the University of Gothenburg, Sweden.

Assessment of sequence variation

To assess sequence variation, six individuals, representing major lineages within *Medicago* and *Melilotus* were chosen. These included the reference sequence (*M. truncatula* Gaertn. HM001 from http://medicagohapmap.org), *M. italica* (Mill.) Grande (PI577295, GRIN), *M. sativa* L. subsp. *sativa* (*M. sativa* hereafter) (PI220598, GRIN), *M. medicaginoides* (Retz.) E. Small (W624116, GRIN) and sequences from two species of *Melilotus* (*Melilotus sulcatus* Desf. and *Melilotus neapolitanus* Ten. ex Guss., both provided as seed by Sienna Botanic Garden). Raw sequence reads were deposited in the European Nucleotide Archive with the following sample accession numbers: *M. italica*: ERS511665; *M. sativa* subsp. *sativa*: ERS511666; *M. medicaginoides*: ERS511669; *Melilotus sulcatus*: ERS511667; *Melilotus neapolitanus*: ERS511668.

Assemblies were performed with the CLC Genomics software (CLC Bio, Aarhus, Denmark). Reads were stripped of adapter sequences and filtered for quality with a threshold of 20 for phred-scores. Different assembly parameters were tested for each sample to increase contig length, the best results being attained with a word size of 30 to 40 and a bubble size of 900. To retrieve on-target contigs (assembled reads), a BLAST database was created for each sample. Each database was queried against each probe gene sequence and homologous sequences were retrieved at E-value $\leq$ 1E-100. Finally, homologous sequences were aligned using Mafft v7.123 [46] with the following settings:—adjustdirection—maxiterate 1000—op 10—globalpair. Each individual alignment was refined in Geneious Pro v.5.3.6 (Biomatters Ltd.) using the Geneious alignment option with default parameters, and edited manually to minimise potential false homologies. Spurious sequences or segments, i.e., missassembled or non-orthologous sequences, were removed from the alignments. In each locus, contigs belonging to the same individual were merged into a single consensus sequence. Overlapping segments of contigs were merged and variants were coded according to the IUPAC standard using methods from the Pycogent [47] and Biopython [48] libraries. Scripts used for retrieving sequences and merging contigs are available upon request.

Model testing was performed on each gene in JModelTest v. 2.1.4 [49–50]. The number of parsimony-informative sites, as well as a maximum likelihood (ML) tree, inferred with the corresponding model of substitution, the two *Melilotus* species as outgroup, and with a 500 replicate bootstrap analysis, were obtained for each alignment of six sequences using PAUP* v.4.0b10 [51]. The average of patristic distances between taxa on either side of the *Medicago*/*Melilotus* split (i.e., across the root) was scored for each gene tree in Geneious Pro (v.5.3.6). As the age of the split between the two genera has been estimated at 15.9 Ma [27], [30], and since patristic distances correspond to branch lengths in substitutions [52], it was possible to estimate substitution rates for each marker. The distributions of rates for the combined set of loci were plotted as histograms and curve fitted to normal and gamma distributions using

likelihood functions in R [53]. Evaluations of which functions best represent the underlying distribution of the 50 rates were assessed by visual comparisons of quantile-to-quantile (qq) plots for the normal and gamma distributions. Analyses were performed on the bioinformatics computer cluster Albiorix at the Department of Biological and Environmental Sciences, University of Gothenburg.

Results

We successfully captured and aligned 50 new LCN genes in our sample. Recovery of contigs through our bioinformatic protocol failed for two of the 62 selected genes. The causes of this failure are unclear since no differences in terms of size, proportion of exon and intron, GC content, or expression levels were detected between these two genes and the remaining 60. Reads could be mapped to the reference sequence of these two genes, but not in enough quantity to produce usable sequences. Ten other markers were not considered successfully recovered for one of several reasons, either that the contigs were too short and did not span the full length of the target, or the contigs were not alignable, or the alignments included sections that may have paralogous origins in some sequences. The unsuccessful contig recovery in these genes appears to be, in some cases, related to the phylogenetic distance from the reference species. In total, 12 of the 62 targeted genes were discarded from subsequent analyses. The 50 remaining genes and their respective sizes, parsimony-informative sites, G-C contents and approximate substitution rates are reported in Table 1.

The estimated substitution rates across the 50 genes are distributed across a limited range and appear to be normally distributed with mild indication of skewness. Given the average patristic distances between *Medicago* and *Melilotus* species ranging from 0.06 to 0.19 (mean 0.12), and the estimated age of root node (15.9 Ma: [27], [30]), the range of rates are distributed between 1.9E-9 and 6.1E-9 (mean 3.6E-9) substitutions per site per year (Fig. 1A).

ML analysis inferred three alternative topologies among the 50 gene trees. The outgroup (*Melilotus sulcatus* and *Melilotus neapolitanus*) was recovered as monophyletic in all trees. The clade of *M. truncatula-M.italica* was also present in all trees. The placement of *M. sativa* and *M. medicaginoides* varied between trees, but *M. sativa* was found to be sister to the *M. truncatula-M. italica* clade in the majority of cases (Topology 1, Fig. 2). This arrangement (Topology 1) was supported ($\geq$ 80% BS) by 34 loci, whereas Topology 2 and Topology 3 were supported by only two loci and one locus, respectively (Fig. 2). Thus, 74% of alignments allowed the inference of gene phylogenies supported at all nodes. The sequences used for probe design as well as the alignments obtained for each locus are available as S1 Probe Sequences and S1 Alignments.

Discussion

Sequence-capture

From our initial sampling of 62 LCN, 50 markers were successfully captured in species of both *Medicago* and *Melilotus* (L.) Mill., another genus of tribe Trifoliae, subtribe Trigonellinae, which corresponds to c. 80% of recovery success. These results indicate that when performing sequence-capture across multiple species the initial target should be large enough that the failure to recover a proportion of it does not compromise the utility of the resulting data. There were no significant differences in the total number of reads between samples of *Medicago* and *Melilotus*, which suggests that probes are equally efficient in both genera, Sequence capture using DNA extracted from herbarium material, both in the sequencing run described here and in subsequent runs using our probe set (see additional methods in S1 File), yielded results equivalent to those generated from DNA extractions from silica dried leaf tissue. Alignments produced with the sequences generated ranged from 1806 to 3168 bp in length, which

Table 1. The 50 loci proposed with the respective linkage group, gene reference in Mt3.0 annotation, captured portion of the gene (bp positions in the reference sequence), alignment length, substitution rate ([subst./site/year] E-9),G-C content, number and % of parsimony-informative (PI) sites, % of exon based on the reference sequence, Consistency index (CI) and Retention index (RI).

locus	linkage group	gene reference (Mt3.0)	portion captured (bp)	alignment length	rate [subst/site/y](E-9)	% GC	PI sites	% PI sites	% exon	CI	RI
1	L1	Medtr1g046610.1	961–3210	2368	5.25	28.5	153	6.46	24.22	0.97	0.93
2	L1	Medtr1g046590.1	2198–4140	2103	3.31	28.4	102	4.85	35.77	0.96	0.89
3	L1	Medtr1g046550.2	2881–5641	2883	2.62	33.6	152	5.27	37.12	0.96	0.91
4	L1	Medtr1g046580.1	76–2068	2394	6.10	34.2	133	5.56	35.22	0.95	0.83
5	L2	Medtr2g038160.1	1450–3600	2437	3.18	30.6	143	5.87	34.77	0.98	0.97
6	L2	Medtr2g038230.1	8–2737	3168	3.97	35.4	177	5.59	46.81	0.92	0.77
7	L2	Medtr2g038270.1	2448–4790	2445	2.68	32.7	120	4.89	35.08	0.99	0.97
8	L3	Medtr3g079820.1	1166–3496	2518	3.85	34.1	150	5.96	41.06	0.98	0.95
9	L3	Medtr3g079830.1	2122–4386	2366	3.16	36.4	141	5.96	37.09	0.97	0.94
10	L3	Medtr3g079840.1	4606–7039	2630	3.50	30.5	172	6.54	35.25	0.96	0.91
11	L4	Medtr4g092600.1	3041–5760	3048	4.01	33.5	187	6.14	32.35	0.98	0.96
12	L4	Medtr4g092630.3	1601–3887	2406	3.71	34.2	197	8.19	49.80	0.96	0.93
13	L4	Medtr4g092670.1	401–2877	2583	3.45	34.7	172	6.66	20.39	0.97	0.94
14	L5	Medtr5g033490.1	1505–4516	3158	3.24	34.6	190	6.02	47.01	0.95	0.89
15	L6	Medtr6g017950.1	935–3057	2472	5.14	34.3	189	7.65	33.25	0.95	0.86
16	L6	Medtr6g017970.1	5094–7760	2807	3.64	31.8	169	6.02	28.31	0.95	0.89
17	L7	Medtr7g064560.1	128–2882	2896	3.92	33.7	140	4.83	37.35	0.95	0.83
18	L7	Medtr7g064580.1	539–2753	2450	5.30	36	170	6.94	38.19	0.94	0.84
19	L8	Medtr8g083970.1	4729–7255	2665	1.90	33	93	3.49	35.77	0.98	0.95
20	L8	Medtr8g084020.1	2881–5420	3068	3.39	31.5	105	3.42	26.93	0.95	0.82
21	L8	Medtr8g084050.1	1423–3498	2236	3.18	32.2	130	5.81	34.10	0.97	0.93
22	L9	Medtr3g010000.1	2641–4815	2406	3.72	27.4	81	3.37	42.25	0.94	0.81
23	L9	Medtr3g009920.1	1681–4302	2839	4.83	31.7	208	7.33	40.20	0.96	0.89
24	L10	Medtr5g091180.1	401–3043	3037	4.31	28	101	3.33	39.88	0.98	0.92
25	L10	Medtr5g091210.1	1422–3771	2655	4.83	34.4	203	7.65	39.66	0.95	0.89
26	L10	Medtr5g091250.1	131–2249	2284	3.74	30.3	119	5.21	22.09	0.97	0.91
27	L11	Medtr6g083920.1	1–2318	2384	3.05	36.8	149	6.25	66.78	0.96	0.91
28	L11	Medtr6g083950.1	1841–4187	2657	4.69	33.5	168	6.32	31.57	0.95	0.88
29	L12	Medtr4g114440.1	1024–2897	1937	2.72	36.3	119	6.14	55.18	0.97	0.95
30	L12	Medtr4g114500.1	1281–3680	2482	4.00	34.9	207	8.34	37.00	0.94	0.88
31	L13	Medtr7g088900.1	1410–3844	2478	2.02	35.8	104	4.20	41.85	0.94	0.83
32	L13	Medtr7g088910.1	858–2983	2161	3.49	33.9	148	6.85	33.11	0.96	0.92
33	L13	Medtr7g088950.1	7521–9840	2465	2.44	33.7	95	3.85	40.22	0.96	0.86
34	L14	Medtr1g120740.1	3525–6095	2754	3.49	34.2	168	6.10	49.09	0.95	0.86
35	L14	Medtr1g120720.1	1841–3553	1821	3.51	32	105	5.77	32.69	0.97	0.94
36	L14	Medtr1g120640.1	2177–4419	2341	2.44	31.2	98	4.19	33.35	0.94	0.83
37	L15	Medtr2g122490.1	4793–6770	2056	2.78	32.6	101	4.91	28.72	0.97	0.92
38	L15	Medtr2g122410.1	4081–6431	2430	2.83	31.5	142	5.84	28.63	0.95	0.90
39	L15	Medtr2g122350.1	3766–5696	1998	3.26	31.9	135	6.76	36.82	0.97	0.93
40	L16	Medtr7g005780.1	1197–3112	2076	3.75	33.5	122	5.88	46.92	0.95	0.88
41	L16	Medtr7g005790.1	1231–3476	2367	4.73	34.1	113	4.77	25.20	0.94	0.76
42	L17	Medtr8g025590.1	1085–3065	2042	3.22	31.8	123	6.02	38.31	0.99	0.97
43	L17	Medtr8g025650.1	603–2372	1806	3.73	35.1	132	7.31	36.33	0.97	0.96
44	L18	Medtr2g046770.1	1521–3997	2830	5.70	34.2	254	8.98	39.89	0.95	0.90
45	L18	Medtr2g046700.1	2732–5144	2795	4.11	34.9	190	6.81	36.51	0.95	0.87

(*Continued*)

Table 1. (*Continued*)

locus	linkage group	gene reference (Mt3.0)	portion captured (bp)	alignment length	rate [subst/site/y](E-9)	% GC	PI sites	% PI sites	% exon	CI	RI
46	L18	Medtr2g046560.1	1321–3086	1833	2.24	33.1	93	5.07	29.73	0.99	0.98
47	L19	Medtr4g080820.1	641–2957	2412	3.72	35.4	144	5.97	48.25	0.97	0.94
48	L20	Medtr3g113790.1	8490–10497	2137	2.23	33.6	94	4.40	33.62	1.00	0.99
49	L20	Medtr3g113840.1	5320–7261	1980	4.47	34.5	101	5.10	36.20	0.96	0.91
50	L20	Medtr3g113960.1	2051–3847	1886	2.01	32.2	75	3.98	37.34	0.97	0.93

doi:10.1371/journal.pone.0109704.t001

corresponds to a significant overall increase in sequence length and consequently to more informative data compared to other methods (e.g., RAD-tag sequencing). In addition to genes

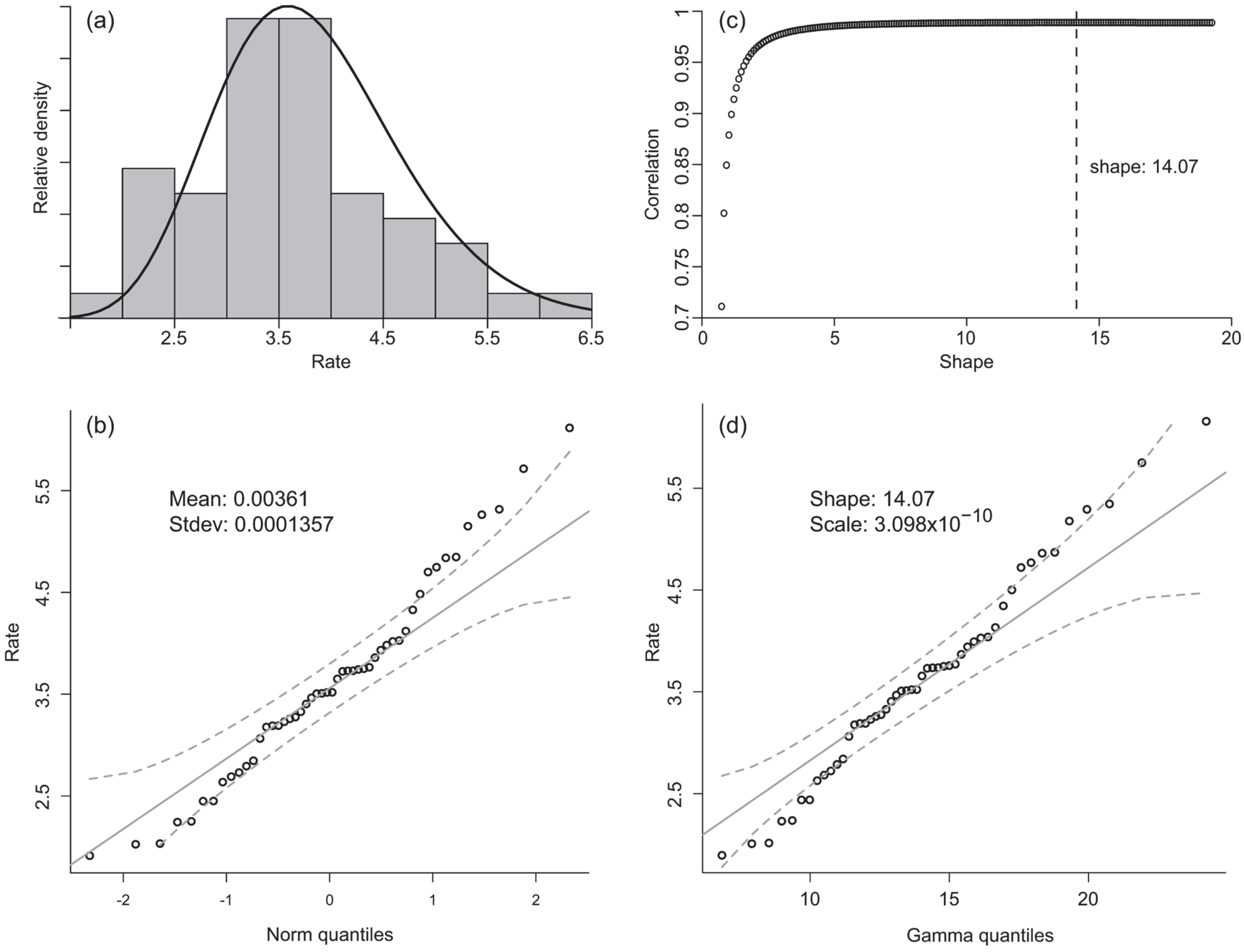

Fig 1. Substitution rates for the 50 nuclear genes. a) Histogram of rates plotted for 50 low copy nuclear genes, fitted with a gamma distribution curve. b) Quantile—Quantile plot describing the fit of 50 nuclear gene rates to a normal distribution. Solid line represent expected values under assumption of perfect normality, with dashed lines indicating the 95% credibility interval. c) Probability plot correlation coefficient (PPCC) plot of shape parameter for a gamma distribution fit to 50 low copy nuclear genes. Optimal shape value indicated by dashed line. d) Quantile—Quantile plot describing the fit of 50 nuclear gene rates to a gamma distribution under the optimised shape value (Fig. 1C). Solid line represent expected values under assumption of perfect fit to a distribution with scale = 3.098E-10 and shape = 14.07. Dashed lines indicate boundaries of the 95% credibility interval.

doi:10.1371/journal.pone.0109704.g001

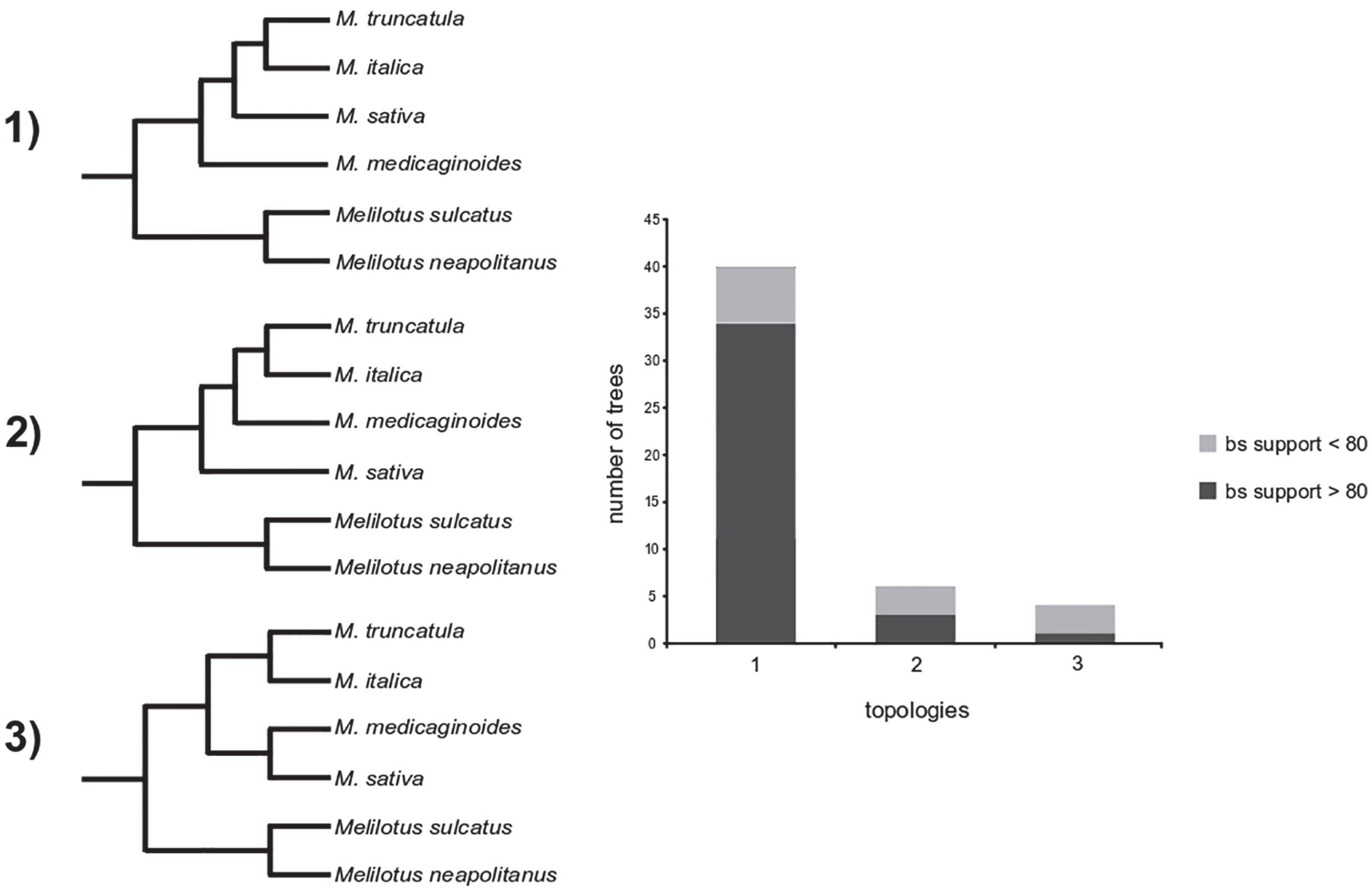

Fig 2. Frequency of recovery of each gene-tree topology across 50 genes and proportion of trees with bootstrap support greater than 80%.

doi:10.1371/journal.pone.0109704.g002

that were purposely captured, and possibly due to the modest size of our target, contigs that match plastid sequences were also obtained from our pools of reads, which corroborates previous findings on targeted enrichment combined with genome skimming [17], but no homology assessment was done for these sequences as it was not the purpose of the present contribution. Our results show how sequence capture can be efficiently used to generate data for multiple loci and species/individuals in a phylogenetic study at the genus or tribe level using a single reference genome. Multiplexing of eight individuals in each sequence capture reaction, and 48 individuals in the sequencing reaction resulted in significantly reduced costs and labour time compared to a similar data set generated through amplicon sequencing.

Gene rate variation

The estimated mean substitution rate for the 50 *Medicago* genes (3.6E-9) is slightly lower than the average rate found for 10 examples of ITS (internal transcribed spacer) rates in herbaceous plants (4.1E-9, [54]). Depending on the selection of genes, the average aligned length of *Medicago* sequences (2.5 kb) can contain nearly three times the information when compared to ITS (c. 0.7 kb). The c. 3-fold difference between the lowest and highest rates estimated from the 50 genes agrees with previous observations based on K_s (synonymous substitutions) in plant nuclear genes [55–56].

This observation suggests that rate variation in expressed LCN genes is distributed within predictable boundaries that can be used for alignments of other LCN genes, thus providing

informative prior knowledge regarding substitution rates for use in Bayesian methods of phylogenetic reconstruction and/or molecular dating. When used, such information will make it possible to estimate divergence times for groups where explicit or reliable knowledge for calibration of the molecular clock is sparse, dubious, or even lacking (e.g., fossils or geological events). It must be noted, however, that such an assumption relies on the distribution of rates presented here being, in turn, derived from reliable and objective use of fossil or geological information.

Also, rather than modelling rate variation in a Bayesian framework by using flat or uninformative priors (i.e., a uniform distribution with hard upper and/or lower boundaries), our results show a more reasonable approach to definition and limitation of the prior parameter space. The use of informative priors over uninformative ones is likely to increase the precision of marginal likelihood estimates [57], and is of special concern when using marginal likelihoods for Bayes Factor comparisons [58]. Comparing the distribution of rates in this study to similar studies based on ITS rates for plants with different life histories [54], or more generally [59–60], leads us to the conclusion that a plausible prior range of substitution rates appears to be neither impossible to define nor infinite. As *Medicago* includes both perennial (*M. sativa*) and annual (*M. truncatula*, *M. italica*, *M. medicaginoides*) herbs, as well as woody species, we believe that our results can be extrapolated to plants with a wide range of growth habits.

Here, we suggest the use of the distribution inferred from our 50 loci as prior information for markers with similar properties. To avoid imposing overly strict limits on the variation (hard boundaries), we suggest that the prior probabilities are best defined using dual open-ended distributions, such as the normal or gamma distributions. For the 50 rates presented in this study, a gamma distribution appears slightly better at describing the underlying rate distribution than would a normal, but the relative fit may vary as information from more genes become available. Although a normal distribution with mean 3.611E-9 and a standard deviation of 1.357E-10 captures much of the variation (Fig. 1B), a gamma distribution with shape 14.076 (Fig. 1C) and scale 3.098E-10 (Fig. 1D) may be a better fit under the assumption of skewness in the distribution.

It must be noted however, under the assumption of all rates for this study being derived from fossil data, that many rates may in fact be overestimated if the age of the fossil record used for calibration is underestimated. Also, rates may be overestimated if the alignments contain misidentified paralogues. Assuming the underlying distribution is normal, such overestimates may be compensated for by increasing the standard deviation. However, due to the relationship between shape and scale parameters, there are no simple means to compensate for increased uncertainty due to overestimated rates in a gamma distribution. We therefore encourage the readers to see the above values, for standard deviations, shape and scale, primarily as guidelines to make informed choices for the datasets on which they are applied. It is also necessary to point out that, under certain conditions where multiple loci share the same tree (e.g. when concatenating 30 or more genes) and the rate variance becomes close to zero, using a misspecified gamma or lognormal rate prior may lead to incorrect divergence time estimates, thus caution should be taken [61].

Topological variation

As expected from other studies (e.g., [62]), we found topological variation among the 50 genes we analysed. These different topologies illustrate how phylogenetic incongruence in *Medicago* is found even at deep nodes with relatively old divergence times. Both the outgroup and the clade containing *M. truncatula* and *M. italica* were always recovered with bootstrap support >95%. In contrast, *M. medicaginoides* and *M. sativa* show alternative placements in the

different phylogenies, albeit with a clear predominant topology (topology 1) which is compatible with the earlier ITS [31], [33] and chloroplast trees [28–29]. The branch length between the clades containing *M. medicaginoides* and *M. sativa*, based on a calibrated nuclear ITS phylogeny, is estimated at c. 6 My (Pfeil unpublished data). In terms of coalescent time, the probability of incomplete lineage sorting (ILS) explaining the observed alternative branching of either taxa is low, unless very large population sizes are considered. This is not expected according to previous results [30], and indicates that other sources of incongruence that have been hypothesised previously, such as introgression or paralogy (*loc. cit.*) may be the cause of the other two topologies inferred.

These results show that alternative well-supported gene trees can be obtained from our sampled genes, which in turn opens the way of uncovering the processes behind the incongruence observed in earlier phylogenies, particularly at the deeper nodes of the gene trees. Comparing linked and unlinked genes may also contribute to identify different causes of incongruence. Genes affected by processes such as ILS and introgression can be used for phylogenetic inference using current methods, but these processes must necessarily be identified prior to species tree reconstruction. From our sample of six individuals we cannot ascertain whether all species relationships in genus *Medicago* will be solved using the proposed genes, which is, in any case, not the subject of the present contribution. However, several of our genes were used to investigate the relationships and putative reticulation between a number of closely related species, using multiple individuals per species (Eriksson et al., in prep; see S1 File for methods and figures), and showed that shallow relationships in *Medicago* could be resolved with high support, which is a prerequisite for distinguishing congruence and conflict [37].

Further use of the proposed markers

As an alternative to the continuing practice of primer development for amplification of targeted markers by PCR (e.g., [63–64]), we demonstrate the use of sequence-capture and NGS in *Medicago* and obtain data from 50 markers that allow gathering adequate phylogenetic data for the clade at hand and potentially for other plant groups. Primers normally used in PCR methods are vulnerable to mutations at the priming site, such that a single change at the 3' end may be enough to render them unusable across different species. In contrast, the tiling method used in sequence capture is not as sensitive to such mutations. All sequences used here for probe design are made available as a resource for further use in sequence-capture in *Medicago*, *Melilotus* and *Trigonella*. Another group with a poorly-resolved phylogeny where our set of genes may be applied include tribe Vicieae, which comprises genera of high agricultural importance such as *Lens* and *Pisum* [28].With some adjustment of the sequence capture conditions, such as hybridisation temperature, gene recovery in very distant lineages is also possible [19]. However, it should be noted that our use of exon and intron probes, based on the *M. truncatula* genome sequence, probably accounted for an increased efficiency of intron capture—a luxury not available in every taxonomic group. Despite this, exon-only probes are known to perform well in any case, successfully capturing flanking introns [22], making the transfer to other groups likely to succeed. The main challenge would be to capture and assemble long introns, but using long fragment sizes relative to intron sizes (perhaps at least as long) may facilitate intron-spanning assembly with the use of exon-only probes.

The alignments provided may also be used to incorporate the observed variation into probe design. Alternatively, the most conserved portions in these alignments can be used for primer design to amplify specific portions of the loci, if desired. Further sampling of these same genes in more distantly related legumes will allow for the inclusion of yet greater variation in probe design, making probes efficient across a larger number of samples, and may contribute to the

development of family-wide sets of probes for use in extended phylogenetic studies, similar to what has been done for family Asteraceae [15].

Given the currently available sequence capture and NGS technology, obtaining and testing a large number of loci for phylogenetics has become much easier. However, since not all genes have the desired properties for phylogenetics [11], capturing genes that have already been tested and reviewed for phylogenetic performance could increase the proportion of useful data in a dataset, and thus improve the efficiency of these methods. The use of common markers also allows for orthologous comparisons to be made across different groups, helping to solve phylogenetic questions at higher taxonomic levels, which have, so far, been mostly based on chloroplast markers [65].

Conclusions

The multiplex approach followed here proved to be cost- and labour-effective if compared to PCR-based approaches, especially considering the amount and properties of the data produced.

We demonstrated that probes designed from *Medicago truncatula* are efficient for targeted capture of nuclear genomic DNA, including variable introns, in two genera (*Medicago* and *Melilotus)* of tribe Trifoliae that diverged over 15 Ma ago. All 50 loci proposed have sequence length and variation that is suitable for gene tree inference and can be sequenced using the probe set provided here together with sequence-capture enrichment methods. Sequences obtained for *Medicago* and *Melilotus*, and the assessment of variation for each gene, may also become useful for studies in other genera within the large, economically and ecologically important legume family. The data provided in this study can serve as template for choice of markers and probe design, and the assessment of substitution rate variation among plant LCN provides prior information for analyses of datasets with similar properties.

Supporting Information

S1 Alignments. Folder containing alignments of 50 loci; each alignment is identified by the same name as in the probe sequence file (e.g., gene46_Medtr2g046560.1).
(ZIP)

S1 File. Figure A, Calibrated BEAST trees of gene 7 showing posterior probability values for each node. Figure B, Calibrated BEAST trees of gene 9 showing posterior probability values for each node. Figure C, Calibrated BEAST trees of gene 48 showing posterior probability values for each node. Figure D, Calibrated BEAST trees of gene 50 showing posterior probability values for each node. Methods A, Additional methods. Table A, Reads per sample before and after adapter removal and quality trim (phred score threshold of 20).
(PDF)

S1 Probe Sequences. File containing all sequences used as probes for sequence-capture of 50 loci.
(FAS)

Acknowledgments

We thank several anonymous reviewers for very constructive comments that greatly improved this manuscript.

Author Contributions

Conceived and designed the experiments: FS BO BEP. Performed the experiments: FS JSE. Analyzed the data: FS JSE SN. Contributed reagents/materials/analysis tools: YJKB. Wrote the paper: FS BO BEP JSE SN YJKB.

References

1. Cronn R, Knaus BJ, Liston A, Maughan PJ, Parks M et al. (2012) Targeted enrichment strategies for next-generation plant biology. American Journal of Botany 99: 291–311. doi: 10.3732/ajb.1100356 PMID: 22312117
2. Guschanski K, Krause J, Sawyer S, Valente LM, Bailey S et al. (2013) Next-Generation Museomics Disentangles One of the Largest Primate Radiations. Systematic Biology 62: 539–554. doi: 10.1093/sysbio/syt018 PMID: 23503595
3. Eaton DAR, Ree RH (2013) Inferring Phylogeny and Introgression using RADseq Data: An Example from Flowering Plants (*Pedicularis*: Orobanchaceae). Systematic Biology 62: 689–706. doi: 10.1093/sysbio/syt032 PMID: 23652346
4. Carstens BC, Brennan RS, Chua V, Duffie CV, Harvey MG et al. (2013) Model selection as a tool for phylogeographic inference: an example from the willow *Salix melanopsis*. Molecular Ecology 22: 4014–4028. doi: 10.1111/mec.12347 PMID: 23848064
5. Smith BT, Harvey MG, Faircloth BC, Glenn TC, Brumfield RT (2014) Target capture and massively parallel sequencing of ultraconserved elements for comparative studies at shallow evolutionary time scales. Systematic Biology 63: 83–95. doi: 10.1093/sysbio/syt061 PMID: 24021724
6. McCormack JE, Hird SM, Zellmer AJ, Carstens BC, Brumfield RT (2013) Applications of next-generation sequencing to phylogeography and phylogenetics. Molecular Phylogenetics and Evolution 66: 526–538. doi: 10.1016/j.ympev.2011.12.007 PMID: 22197804
7. Schneeberger K, Ossowski S, Ott F, Klein JD, Wang X et al. (2011). Reference-guided assembly of four diverse *Arabidopsis thaliana* genomes. Proceedings of the National Academy of Sciences 108: 10249–10254. doi: 10.1073/pnas.1107739108 PMID: 21646520
8. Lam H, Xu X, Liu X, Chen W, Yang G et al. (2010) Resequencing of 31 wild and cultivated soybean genomes identifies patterns of genetic diversity and selection. Nat Genet 42: 1053–1059. doi: 10.1038/ng.715 PMID: 21076406
9. Miller MR, Dunham JP, Amores A, Cresko WA, Johnson EA (2007) Rapid and cost-effective polymorphism identification and genotyping using restriction site associated DNA (RAD) markers. Genome Research 17: 240–248. PMID: 17189378
10. Wang XQ, Zhao L, Eaton DAR, Li DZ, Guo ZH (2013). Identification of SNP markers for inferring phylogeny in temperate bamboos (Poaceae: Bambusoideae) using RAD sequencing. Molecular Ecology Resources 13: 938–945. doi: 10.1111/1755-0998.12136 PMID: 23848836
11. Thomson RC, Wang IJ, Johnson JR (2010) Genome-enabled development of DNA markers for ecology, evolution and conservation. Molecular Ecology 19: 2184–2195. doi: 10.1111/j.1365-294X.2010.04650.x PMID: 20465588
12. Straub SCK, Parks M, Weitemier K, Fishbein M, Cronn RC et al. (2012) Navigating the tip of the genomic iceberg: Next-generation sequencing for plant systematics. American Journal of Botany 99: 349–364. doi: 10.3732/ajb.1100335 PMID: 22174336
13. Lemmon AR, Lemmon EM (2012). High-Throughput Identification of Informative Nuclear Loci for Shallow-Scale Phylogenetics and Phylogeography. Systematic Biology 61: 745–761. doi: 10.1093/sysbio/sys051 PMID: 22610088
14. Stull GW, Moore MJ, Mandala VS, Douglas NA, Kates H et al. (2013). A Targeted Enrichment Strategy for Massively Parallel Sequencing of Angiosperm Plastid Genomes. Applications in Plant Sciences 1: 1–7.
15. Mandel JR, Dikow RB, Funk VA, Masalia RR, Staton SE et al. (2014) A Target Enrichment Method for Gathering Phylogenetic Information from Hundreds of Loci: An Example from the Compositae. Applications in Plant Sciences 2. http://dx.doi.org/10.3732/apps.1300085
16. Grover CE, Zhu X, Grupp KK, Jareczek JJ, Gallagher JP et al. (2014) Molecular confirmation of species status for the allopolyploid cotton species, Gossypium ekmanianum Wittmack. Genetic Resources and Crop Evolution 61: 1–12.
17. Weitemier K, Straub SCK, Cronn RC, Fishbein M, Schmickl R et al. (2014) Hyb-Seq: Combining target enrichment and genome skimming for plant phylogenomics. Applications in Plant Sciences 2: in press.

18. Mamanova L, Coffey AJ, Scott CE, Kozarewa I, Turner EH et al. (2010) Target-enrichment strategies for next-generation sequencing. Nature methods 7: 111–118. doi: 10.1038/nmeth.1419 PMID: 20111037
19. Li C, Hofreiter M, Straube N, Corrigan S, Naylor G (2013) Capturing protein-coding genes across highly divergent species. Biotechniques 54: 321–326. doi: 10.2144/000114039 PMID: 23758502
20. Staats M, Erkens R, van de Vossenberg B, Wieringa JJ, Kraaijeveld K et al. (2013) Genomic Treasure Troves: Complete Genome Sequencing of Herbarium and Insect Museum Specimens. PLoS ONE 8: e69189. doi: 10.1371/journal.pone.0069189 PMID: 23922691
21. Enk J, Devault A, Kuch M, Murgha Y, Rouillard J et al. (2014) Ancient whole genome enrichment using baits built from modern DNA. Molecular Biology and Evolution 31: 1292–1294. doi: 10.1093/molbev/msu074 PMID: 24531081
22. Lemmon AR, Emme SA, Lemmon EM (2012) Anchored Hybrid Enrichment for Massively High-Throughput Phylogenomics. Systematic Biology 61: 727–744. doi: 10.1093/sysbio/sys049 PMID: 22605266
23. Egan AN, Schlueter J, Spooner DM (2012) Applications of next-generation sequencing in plant biology. American journal of botany 99: 175–185. doi: 10.3732/ajb.1200020 PMID: 22312116
24. McMahon MM, Sanderson MJ (2006).Phylogenetic Supermatrix Analysis of GenBank Sequences from 2228 Papilionoid Legumes. Systematic Biology 55: 818–836. PMID: 17060202
25. Thomson RC, Shaffer HB (2009) Sparse Supermatrices for Phylogenetic Inference: Taxonomy, Alignment, Rogue Taxa, and the Phylogeny of Living Turtles. Systematic Biology 59: 42–58. doi: 10.1093/sysbio/syp075 PMID: 20525619
26. Small E (2011) Alfalfa and relatives. evolution and classification of Medicago. Ottawa: NRC Research Press. 727 p.
27. Lavin M, Herendeen PS, Wojciechowski MF (2005) Evolutionary Rates Analysis of Leguminosae Implicates a Rapid Diversification of Lineages during the Tertiary. Systematic Biology 54: 575–594. PMID: 16085576
28. Steele K, Wojciechowski M (2003) Phylogenetic analyses of tribes Trifolieae and Vicieae, based on sequences of the plastid gene, matK (Papilionoideae: Leguminosae). Advances in legume systematics Part 10: 355–370.
29. Steele KP, Ickert-Bond SM, Zarre S, Wojciechowski MF (2010) Phylogeny and character evolution in Medicago (Leguminosae): Evidence from analyses of plastid trnK/matK and nuclear GA3ox1 sequences. American Journal of Botany 97: 1142–1155. doi: 10.3732/ajb.1000009 PMID: 21616866
30. Maureira-Butler IJ, Pfeil BE, Muangprom A, Osborn TC, Doyle JJ (2008) The Reticulate History of Medicago (Fabaceae). Systematic Biology 57: 466–482. doi: 10.1080/10635150802172168 PMID: 18570039
31. Downie SR, Katz-Downie DS, Rogers EJ, Zujewski HL, Small E (1998) Multiple independent losses of the plastid rpoC1 intron in Medicago (Fabaceae) as inferred from phylogenetic analyses of nuclear ribosomal DNA internal transcribed spacer sequences. Canadian Journal of Botany 76: 791–803.
32. Bena G, Jubier M, Olivieri I, Lejeune B (1998) Ribosomal External and Internal Transcribed Spacers: Combined Use in the Phylogenetic Analysis of Medicago (Leguminosae). Journal of Molecular Evolution 46: 299–306. PMID: 9502673
33. Bena G (2001) Molecular phylogeny supports the morphologically based taxonomic transfer of the "medicagoid" Trigonella species to the genus Medicago L.. Plant Systematics and Evolution 229: 217–236.
34. Yoder JB, Briskine R, Mudge J, Farmer A, Paape T et al. (2013) Phylogenetic Signal Variation in the Genomes of Medicago (Fabaceae). Systematic Biology 62: 424–438. doi: 10.1093/sysbio/syt009 PMID: 23417680
35. Sang T (2002) Utility of low-copy nuclear gene sequences in plant phylogenetics. Critical Reviews in Biochemistry and Molecular Biology 37: 121–147. PMID: 12139440
36. Small RL, Ryburn JA, Cronn RC, Seelanan T, Wendel JF (1998) The tortoise and the hare: choosing between noncoding plastome and nuclear Adh sequences for phylogeny reconstruction in a recently diverged plant group. American Journal of Botany 85: 1301–1315. PMID: 21685016
37. Hughes CE, Eastwood RJ, Bailey CD (2006) From famine to feast? Selecting nuclear DNA sequence loci for plant species-level phylogeny reconstruction. Philosophical Transactions of the Royal Society B: Biological Sciences 361: 211–225. PMID: 16553318
38. Cronn R, Cedroni M, Haselkorn T, Grover C, Wendel JF (2002) PCR-mediated recombination in amplification products derived from polyploid cotton. Theoretical and Applied Genetics 104: 482–489. PMID: 12582722

39. Small RL, Cronn RC, Wendel JF (2004) LAS JOHNSON REVI42EW No. 2 Use of nuclear genes for phylogeny reconstruction in plants. Australian Systematic Botany 17: 145–170.
40. Álvarez I, Wendel J (2003) Ribosomal ITS sequences and plant phylogenetic inference. Molecular Phylogenetics and Evolution 29: 417–434. PMID: 14615184
41. Källersjö M, Albert VA, Farris JS (1999) Homoplasy increases phylogenetic structure. Cladistics 15: 91–93. PMID: 10029988
42. Lopez P, Casane D, Philippe H (2002) Heterotachy, an important process of protein evolution. Molecular Biology and Evolution 19: 1–7. PMID: 11752184
43. Kolaczkowski B, Thornton JW (2004) Performance of maximum parsimony and likelihood phylogenetics when evolution is heterogeneous. Nature 431: 980–984. PMID: 15496922
44. Stefanovic S, Pfeil BE, Palmer JD, Doyle JJ (2009) Relationships among phaseoloid legumes based on sequences from eight chloroplast regions. Systematic Botany 34: 115–128.
45. Young ND, Debelle F, Oldroyd GED, Geurts R, Cannon SB et al. (2011) The Medicago genome provides insight into the evolution of rhizobial symbioses. Nature 480: 520–524. doi: 10.1038/nature10625 PMID: 22089132
46. Katoh K, Standley DM (2013) MAFFT multiple sequence alignment software version 7: improvements in performance and usability. Molecular biology and evolution 30: 772–780. doi: 10.1093/molbev/mst010 PMID: 23329690
47. Knight R, Maxwell P, Birmingham A, Carnes J, Caporaso JG et al. (2007) PyCogent: a toolkit for making sense from sequence. Genome Biol 8: 1–16.
48. Cock PJ, Antao T, Chang JT, Chapman BA, Cox CJ et al. (2009) Biopython: freely available Python tools for computational molecular biology and bioinformatics. Bioinformatics 25: 1422–1423. doi: 10.1093/bioinformatics/btp163 PMID: 19304878
49. Darriba D, Taboada GL, Doallo R, Posada D (2012) jModelTest 2: more models, new heuristics and parallel computing. Nature Methods 9: 772–772. doi: 10.1038/nmeth.2109 PMID: 22847109
50. Guindon S, Gascuel O (2003) A simple, fast, and accurate algorithm to estimate large phylogenies by maximum likelihood. Systematic biology 52: 696–704. PMID: 14530136
51. Swofford DL (2003) PAUP*. Phylogenetic Analysis Using Parsimony (* and Other Methods). Version 4.
52. Fourment M, Gibbs MJ (2006) PATRISTIC: a program for calculating patristic distances and graphically comparing the components of genetic change. BMC Evolutionary Biology 6: 1–5. PMID: 16388682
53. Team RC (2012) R: A Language and Environment for Statistical Computing. Vienna: R Foundation for Statistical Computing. doi: 10.1002/jcc.22917 PMID: 22278855
54. Kay K, Whittall J, Hodges S (2006). A survey of nuclear ribosomal internal transcribed spacer substitution rates across angiosperms: an approximate molecular clock with life history effects. BMC Evolutionary Biology 6: 1–9. PMID: 16388682
55. Wolfe KH, Li WH, Sharp PM (1987) Rates of nucleotide substitution vary greatly among plant mitochondrial, chloroplast, and nuclear DNAs. Proceedings of the National Academy of Sciences 84: 9054–9058. PMID: 3480529
56. Zhang L, Vision TJ, Gaut BS (2002) Patterns of Nucleotide Substitution Among Simultaneously Duplicated Gene Pairs in Arabidopsis thaliana. Molecular Biology and Evolution 19: 1464–1473. PMID: 12200474
57. Chung Y, Rabe-Hesketh S, Gelman A, Liu J, Dorie V (2012) Avoiding boundary estimates in linear mixed models through weakly informative priors. U.C. Berkeley Division of Biostatistics Working Paper Series. Working Paper 284. Available: http://biostats.bepress.com/ucbbiostat/paper284
58. Xie W, Lewis PO, Fan Y, Kuo L, Chen M (2011). Improving marginal likelihood estimation for Bayesian phylogenetic model selection. Systematic Biology 60: 150–160. doi: 10.1093/sysbio/syq085 PMID: 21187451
59. Smith S, Donoghue M (2008) Rates of molecular evolution are linked to life history in flowering plants. Science 322: 86–89. doi: 10.1126/science.1163197 PMID: 18832643
60. Lancaster L (2010) Molecular evolutionary rates predict both extinction and speciation in temperate angiosperm lineages. BMC Evol Biol 10: 162. doi: 10.1186/1471-2148-10-162 PMID: 20515493
61. dos Reis M, Zhu T, Yang Z (2014) The Impact of the Rate Prior on Bayesian Estimation of Divergence Times with Multiple Loci. Systematic Biology 63: 555–565. doi: 10.1093/sysbio/syu020 PMID: 24658316
62. Salichos L, Rokas A (2013) Inferring ancient divergences requires genes with strong phylogenetic signals. Nature 497: 327–331. doi: 10.1038/nature12130 PMID: 23657258

63. Bacon CD, Feltus FA, Paterson AH, Bailey CD (2008) Novel nuclear intron-spanning primers for Arecaceae evolutionary biology. Molecular Ecology Resources 8: 211–214. doi: 10.1111/j.1471-8286.2007.01928.x PMID: 21585759

64. Kepler RM, Rehner SA (2013) Genome-assisted development of nuclear intergenic sequence markers for entomopathogenic fungi of the Metarhizium anisopliae species complex. Molecular Ecology Resources 13: 210–217. doi: 10.1111/1755-0998.12058 PMID: 23286460

65. Doyle JJ (2013) The promise of genomics for a "next generation" of advances in higher-level legume molecular systematics. South African Journal of Botany 89: 10–18.
